# Extract of *Lillium candidum* L. Can Modulate the Genotoxicity of the Antibiotic Zeocin

**DOI:** 10.3390/molecules17010080

**Published:** 2011-12-22

**Authors:** Marcela Kopaskova, Lina Hadjo, Bisera Yankulova, Gabriele Jovtchev, Eliska Galova, Andrea Sevcovicova, Pavel Mucaji, Eva Miadokova, Peter Bryant, Stephka Chankova

**Affiliations:** 1 Department of Genetics, Faculty of Natural Sciences, Comenius University, Mlynska dolina B-1, Bratislava 842 15, Slovakia; 2 Institute for Biodiversity and Ecosystem Research, Bulgarian Academy of Sciences, 2 Gagarin Street, Sofia 1113, Bulgaria; 3 Department of Pharmacognosy and Botany, Faculty of Pharmacy, Comenius University, Odbojarov 10, Bratislava 832 32, Slovakia; 4 School of Biological and Medical Sciences, University of St. Andrews, St. Andrews KY16 9TS, Scotland, UK

**Keywords:** zeocin, *Lilium candidum* L. extract, cytotoxicity, genotoxicity, adaptive response, DSBs rejoining

## Abstract

*Lilium candidum* L. extract (LE) is well known in folk medicine for the treatment of burns, ulcers, inflammations and for healing wounds. This work aims to clarify whether the genotoxic potential of the radiomimetic antibiotic zeocin (Zeo) could be modulated by LE. Our results indicate that LE exerts no cytotoxic, DNA-damaging and clastogenic activity in in *Chlamydomonas reinhardtii*, *Pisum sativum* L. and *Hordeum vulgare* L. test systems over a broad concentration range. Weak but statistically significant clastogenic effects due to the induction of micronuclei and chromosome aberrations have been observed in *H. vulgare* L. after treatment with 200 and 300 μg/mL LE. To discriminate protective from adverse action of LE different experimental designs have been used. Our results demonstrate that the treatment with mixtures of LE and Zeo causes an increase in the level of DNA damage, micronuclei and “metaphases with chromatid aberrations” (MwA). Clear evidence has been also obtained indicating that pretreatment with LE given 4 h before the treatment with Zeo accelerates the rejoining kinetics of Zeo-induced DNA damage in *P. sativum* L. and *C. reinhardtii*, and can decrease clastogenic effect of Zeo measured as frequencies of micronuclei and MwA in *H. vulgare* L. Here, we show for the first time that LE can modulate the genotoxic effects of zeocin. The molecular mode of action strongly depends on the experimental design and varies from synergistic to protective effect (adaptive response–AR). Our results also revealed that LE-induced AR to zeocin involves up-regulation of DSB rejoining in *C. reinhardtii* and *P. sativum* L. cells.

## 1. Introduction

The use of plants and herbs for medicinal purposes is widespread. Today 30–40% of all medicines contain one or more bioactive components derived from plants. Recently much attention has been given to the natural phytochemicals with antioxidant, antibacterial, antimutagenic, antimalarial and anticarcinogenic activities [1,2,3,4,5,6,7,8]. 

*Lilium candidum* L. (*Liliaceae*), the so called “white Madonna lily”, is well known in folk medicine for the treatment of burns, ulcers, inflammations and for healing wounds. *Lilium candidum* L. extract (LE) contains various biologically active compounds [9,10]. As the antimutagenic activity of natural compounds often correlates with antioxidant effects and contents of phytochemical substances from the flavonoids group, our hypothesis is that the LE, which is rich in flavonoids and with pronounced antioxidant activity, could possess bioprotective potential.

An adaptive response (AR) as a nonspecific phenomenon has been observed during last 40 years in many organisms ranging from bacteria up to mammals, including humans. Cells, tissues and organisms can often improve their ability to respond to a specific physical or chemical agent when they have been previously exposed to a small “priming” dose of the same or a similar agent [11,12]. Many different types of damaging agents, including ionizing radiation, heat, oxidative stress, alkylating agents, and heavy metals have been reported to induce an adaptive response. Such a protective response also indicates that the cell, once exposed to a small priming dose of toxin or damaging agent is prepared to deal with a subsequent potentially lethal (“test”) dose of the same agent.

The effects of zeocin (Zeo) on the DNA are detrimental for cells. Zeo, as a member of bleomycin/phleomycin family of glycopeptide antibiotics, is known to bind to DNA and induce oxidative stress in different organisms, like ionizing radiation, producing predominantly single and double strand breaks (SSBs/DSBs), as well as base losses [13,14]. Many defense mechanisms have been evolved to minimalize such effects of oxidative stress, including the AR. Thus, in the green alga *Chlamydomonas reinhardtii* Zeo, as a radiomimetic, has been shown to induce an AR [14]. Chankova *et al.* [13] concluded that Zeo-induced AR in *C. reinhardtii* was a result of the induced acceleration of the rejoining of DSBs.

Despite abundant literature available on the effects of bleomycin, studies on the effect of Zeo in plants are rather limited. Moreover, no information is available as to whether Zeo-induced DNA damage could be modulated using natural plant extracts. Here we report on the cytotoxic and genotoxic effects of the antibiotic zeocin on *H. vulgare* L., and we investigate whether the effects of this genotoxin could be modulated by *Lilium candidum* L. bulb extract on *C. reinhardtii* and *P. sativum* L., as well.

## 2. Results and Discussion

### 2.1. LE and Zeo Cytotoxicity

**Figure 1:**
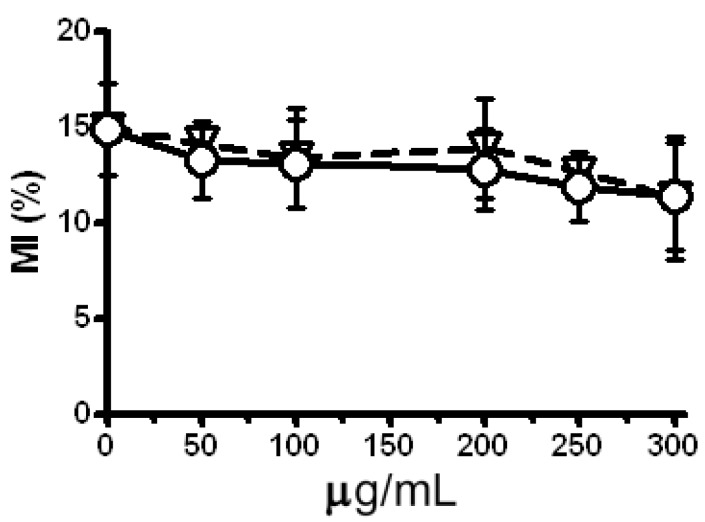
Mitotic index (MI) evaluated after Zeo (triangles) or LE (circles) treatment in *H. vulgare* L. root tips meristem cells. Negative control (untreated sample) is represented by data obtained at zero concentration. Pooled results of three experiments.

The first step of our investigation was to evaluate the cytotoxic potential of LE and Zeo. As a marker for cytotoxicity the mitotic activity of *H. vulgare* L. root tips meristem cells has been analyzed, calculating the mitotic index (MI). Results presented in Figure 1 demonstrate no statistically significant reduction of MI after a single treatment with Zeo or LE compared with the untreated control (P > 0.05). No changes in MI value as a function of concentration could be considered as an indirect evidence for the absence of LE and Zeo cytotoxicity over a broad concentration range. This finding corresponds well with data described by Yarmolinsky *et al.* [15] that no cytotoxicity of *L. candidum* L. bulbs extract has been observed at concentrations below 100 mg/mL and concentrations found to cause 50% toxicity was higher than 500 mg/mL (CC_50_ > 500 mg/mL). 

### 2.2. Zeocin Genotoxicity and Effects of Lilium candidum Extract on *P. sativum* L.

Despite abundant literature available on the action of bleomycin [16,17], studies on the effect of Zeo have been focused on Zeo as causing transformants in various test-systems [18,19,20]. Data on the DNA-damaging action of Zeo in only one plant system has been reported [13,14]. Therefore the second step of this study was to evaluate DNA-damaging (genotoxic) potential of Zeo over a wider concentration range. In previous work using *C. reinhardtii* no significant increase in Zeo-induced DSBs was observed until a concentration of 100 µg/mL [13,14].

**Figure 2:**
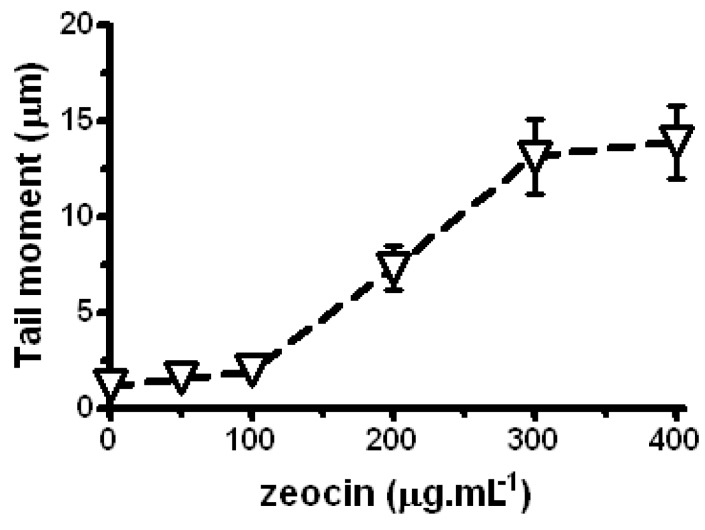
Comet assay data for SSBs and DSBs induced by Zeo in *P. sativum* L*.* Negative control (untreated sample) is represented by data obtained at zero concentration. The error bars represent standard errors of mean values. P < 0.001 between control and Zeo samples by the Student’s t-test. Where no error bars are evident, errors were equal to or smaller than the symbols.

In *P. sativum* L. using the comet assay it has been found that Zeo significantly enhances the level of DNA damage in a dose-dependent manner in the range of 100–300 µg/mL (P < 0.001; Figure 2). In the 300–400 µg/mL range the levels of induced DNA damage (SSBs and DSBs) are approximately constant. In preliminary experiments a plateau occurred even at a Zeo concentration of 600 µg/mL. Our finding concerning the plateau in dose-response curves is in accordance with the results reported by us earlier that Zeo could induce DSBs in *C. reinhardtii* in a linear dose-response fashion up to 100 µg/mL and above this concentration a plateau occurred [13]. Such a plateau has been observed in other test systems using different agents and various theories have been put forth to account for this result [21,22,23]. Because we found no significant difference (P > 0.05) between levels of DNA damage induced above 300 µg/mL we focused our attention in *P. sativum* L. at a concentration of 300 µg/mL.

In *P. sativum* L., like in *C. reinhardti*, the value of DNA damage increases very little as a result of single dose LE-treatment. Data are not shown due to the absence of LE genotoxicity over a broad concentration range (from 50 to 300 μg/mL). The only concentration that exerted genotoxic effects in *P. sativum* L due to a significant increase in SSBs and DSBs, as assessed by the comet assay, is 200 μg/mL. On the basis of these preliminary experiments the concentration of 200 μg/mL has been chosen for assaying the potential modulatory effects of LE.

### 2.3. LE and Zeo Clastogenicity on H. vulgare L.

**Figure 3:**
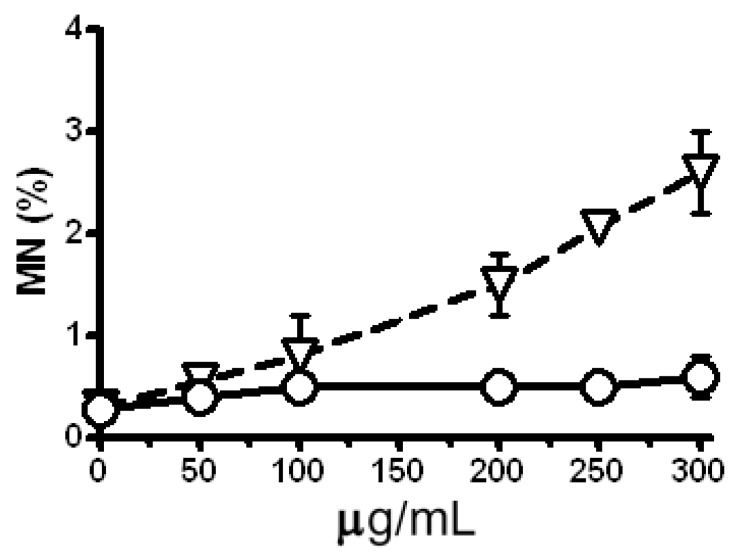
Frequency of micronuclei (MN) in *H. vulgare* L. cells treated with Zeo (triangles) and LE (circles). Negative control (untreated sample) is represented by data at zero concentration. Experiments were carried out in three repetitions for all variants. Where no error bars are evident, errors were equal to or smaller than the symbols.

**Figure 4:**
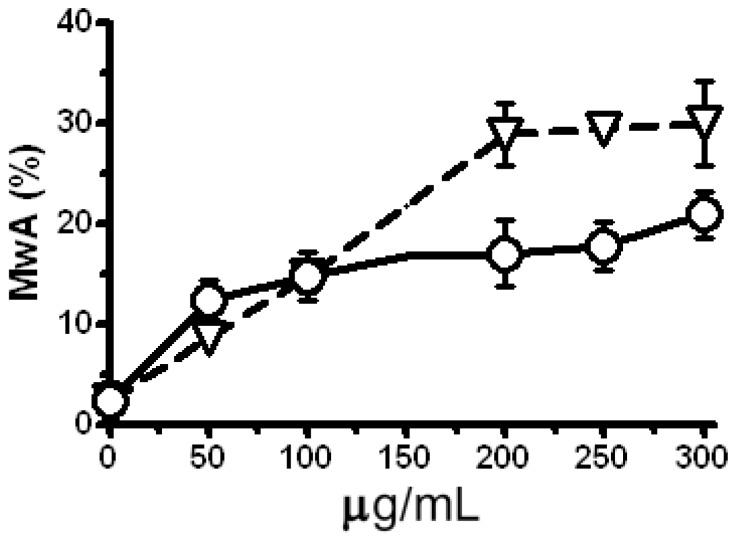
Frequency of metaphases with chromatid aberrations (MwA) induced by Zeo (triangles) and LE (circles) in *H. vulgare* L. A negative control (untreated sample) is represented by data at zero concentration. Experiments were carried out in three repetitions for all variants. All treatment samples showed a significant induction of chromatid aberrations P < 0.001. Error bars represent standard deviation of mean values.

The clastogenic potential of the single dose treatment with LE and Zeo in a test system *H. vulgare* L. was evaluated as a third step of this study. The micronucleus (MN) test detects genetic alternations arising from chromosomal damage and/or damage to the mitotic spindle apparatus caused by clastogenic or aneugenic agents, although the test is less informative than analyses of chromosomal aberrations (the MwA test). It is not clear to which extent specific types of chromosomal rearrangements are reflected by the outcome of the MN. In detail asymmetric translocations, isochromatid breaks, interstitial deletions and spindle defects should lead to MN formation [24,25]. The strong clastogenic effect (P < 0.001) of Zeo is obtained due to the increased level of micronuclei (MN) (Figure 3) and chromatid aberrations in a concentration dependent manner (Figure 4).

LE does not induce a statistically significant enhancement of MN in comparison with the untreated control (Figure 3). After the treatment with concentrations in the 50–300 μg/mL range a statistically significant increase in the level of chromatid aberrations is scored comparing to the control (P < 0.001; Figure 4). On the basis of these experiments the concentrations of 300 μg/mL Zeo and 200 μg/mL, 250 μg/mL and 300 μg/ mL for LE were applied in subsequent experiments.

The lower observed yield of MN in comparison to the chromatid aberrations can be caused by the type of aberrations induced. Having in mind that micronuclei represent a way for the cell to remove some of the resulting fragments [26] the lack of correspondence between the level of MN and MwA could be explained by the type of aberrations. A part of the induced micro-isochromatid breaks located in the terminal regions of the chromosomes could be lost during the mitotic division. 

**Figure 5:**
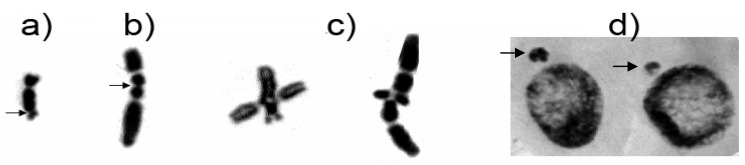
Main types of chromatid aberrations in *H. vulgare* L.: terminal isochromatid breaks after application of LE (**a**); proximal isochromatid breaks after Zeo treatment (**b**); and chromatid translocations induced by Zeo (**c**); and formation of micronuclei (**d**).

Specificity of Zeo and LE clastogenic potential is revealed. The main types of aberrations induced by Zeo are isochromatid breaks, mainly located in the proximal regions of the chromosomes (Figure 5b) and/or chromatid translocations (Figure 5c). On the other hand, isochromatid breaks mainly located in the terminal regions of the chromosomes (Figure 5a) are found after the treatment with LE (200; 250 and 300 μg/mL). 

### 2.4. The Antigenotoxic Effect of Lilium candidum Extract in *C. reinhardtii* and *P. sativum* L.

**Figure 6:**
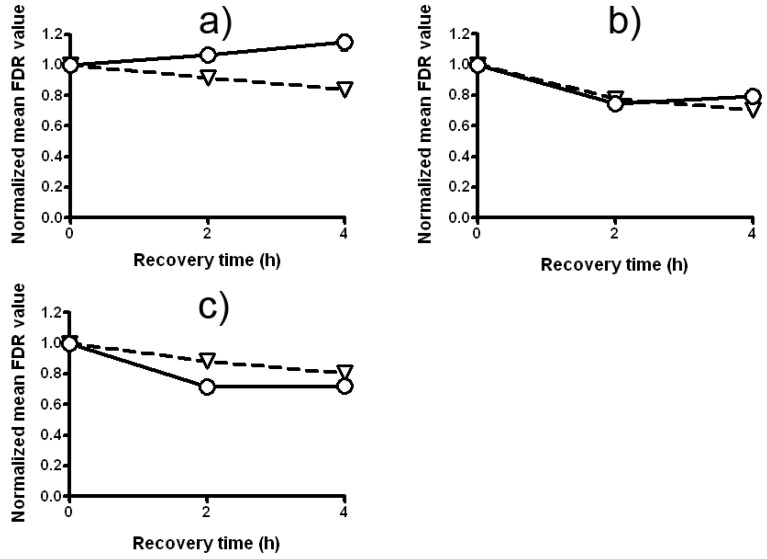
The kinetics of DSB repair in *C. reinhardtii* strain *cw15* depending on the experimental design: (**a**) combined treatment with LE and Zeo (experimental design 1); (**b**) Zeo-treatment given immediately (without intertreatment time) after LE-treatment (experimental design 2); (**c**) Zeo-treatment given 4 h after priming treatment with LE (experimental design 3). Triangles represent Zeo and circles represent LE + Zeo. All experiments were conducted three times with independently grown cell suspensions and P < 0.05. Where no error bars are evident, errors were equal to or smaller than the symbols.

The treatment with a mixture of LE and Zeo (experimental design 1) enhances the mean fraction of DNA damage in *C. reinhardtii.* As it is seen from Figure 6a, the DNA-damaging effects of these two chemicals on *C. reinhardtii* are greater that the effect of Zeo alone. The statistics present that the fraction of damage remaining (FDR) in variants treated with a mixture of LE and Zeo is significantly higher than that in variants treated with a single dose of Zeo (P < 0.001). Strong synergistic DNA-damaging activity is detected when *C. reinhardtii* cells have been treated with a mixture of LE and Zeo. Differences between the two curves are statistically significant (P < 0.01).

Split treatment without any intertreatment time between LE and Zeo causes no statistically significant changes in FDR (experimental design 2) comparing with a variant treated with a single dose of Zeo (Figure 6b). LE pretreatment does not accelerate DSB rejoining (Figure 6b), but also does not increase the DNA-damaging action of Zeo. All data are statistically significant (P < 0.001 and P < 0.001).

Experimental design 3 (split treatment with 2 and/or 4 h intertreatment time between LE- and Zeo-treatment) appears to indicate the potential to protect *C. reinhardtii* by inducing an AR. The fraction of damage remaining after the split treatment with 4 h intertreatment time between LE and Zeo is statistically significantly different from that remaining after single dose Zeo-treatment (P < 0.001). The next question we addressed was whether DSBs up-regulation would be involved in LE-induced AR, similar to radiation- and Zeo-induced AR previously observed by us for *C. reinhardtii* [13,14,27]. For that purpose we measured the kinetics of DSBs rejoining 2 and 4 h after Zeo-treatment with or without LE pretreatment (Figure 6c). The statistical analysis presents clear statistically significant acceleration (P < 0.01) in DSBs rejoining as compared with that received with a single dose of Zeo-treatment. Rejoining of DSBs occurs slowly in none of those pretreated with LE cells. 

**Figure 7:**
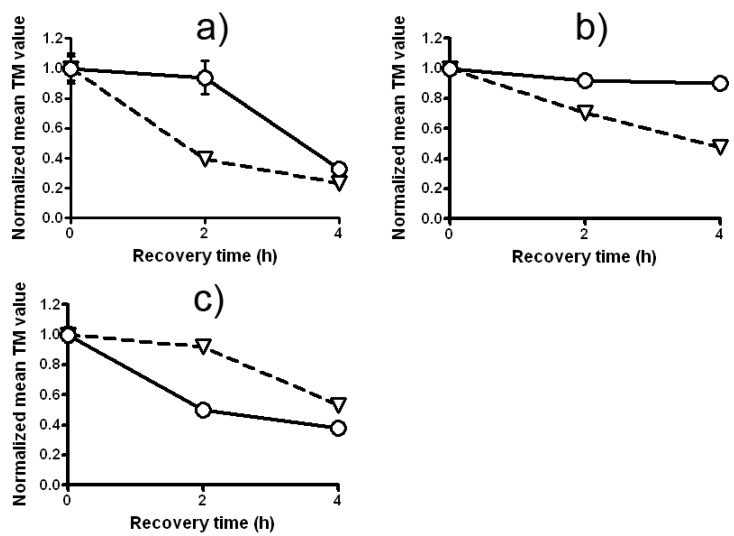
The kinetics of DNA repair in *P. sativum* depending on the experimental design: (**a**) combined treatment with LE and Zeo (experimental design 1); (**b**) Zeo-treatment given immediately after LE-treatment (experimental design 2); (**c**) Zeo-treatment given 4 h after priming treatment with LE (experimental design 3). Triangles represent Zeo and circles represent LE + Zeo. All experiments were conducted in triplicate; P < 0.001. Where no error bars are evident, errors were equal to or smaller than the symbols.

In further experiments a comet assay on a *P. sativum* L. test system was performed. We confirmed our previous finding that LE’s potential to modulate DNA-damaging action of Zeo strongly depends on the experimental design and varies from synergistic to DNA protective. Data in Figure 7a show that the treatment with a mixture of LE and Zeo (experimental design 1) enhances in a statistically significantly way the fraction of damage remaining (SSBs and DSBs) compared to the variant which has received a single Zeo treatment (P < 0.001). Interestingly this synergistic effect is most pronounced 2 h after the treatment and then it becomes smaller.

Very strong synergistic DNA-damaging activity is found in design 2—split treatment without any intertreatment time between LE and Zeo (Figure 7b). Priming treatment with LE strengthens Zeo genotoxicity leading to a clear statistically significant increase of DNA damage as compared with a single Zeo treatment (P < 0.001). Moreover, the level of this synergistic effect is the same 2 and 4 h later (P < 0.001).

The results presented in Figure 7c indicate that pretreatment with LE, given 4 h before the test treatment with Zeo (experimental design 3) induces a strong AR. The fraction of DNA damage remaining in *P. sativum* L. root tips meristem cells. Curves in Figure 7c display that rejoining of DNA damage occurs in both variants, i.e. a decrease with time in the fraction of damage remaining. The level of damage in a pretreated sample is lower than in the sample treated with single Zeo (P < 0.001). 

Thus, our experimental results imply that presence of LE in a mixture enhances the genotoxic and clastogenic effects of Zeo. Such synergistic activity may be due to their different mechanisms of action and might be connected with probabilities of some bio-active LE constituents, e.g., kaempferol and its derivatives to exhibit pro-oxidative activities in the presence of Cu (II) [29,30]. It is generally known that Cu(II) is the component of the Zeo molecule, which is responsible for its radiomimetic properties. Upon copper removal, Zeo is activated, and binds and cleaves DNA, causing cell death. Moreover, kaempferol can exhibit cytotoxic, antiproliferative and apoptosis-inducing properties [31,32]. Genotoxicity enhancement may be also caused by pyrroline-and pyrrolidine alkaloid constituents present in LE. It has been found that pyrroline-and pyrrolidine alkaloids may induce significant DNA damage and oxidative stress, which may even lead to cell death by apoptosis or necrosis [33]. Steroidal saponins were also isolated from the bulbs of *L. candidum* L. [9]. In addition, these LE constituents may change cell membranes fluidity [34] enabling faster Zeo penetration. 

In our split experiments (design 2), without intertreatment time between LE and Zeo, the role of genotype is demonstrated. Pretreatment with LE results in quite different responses in the different plants models used and effects vary from the absence of effects to synergistic and even protective effects.

Strong AR is induced when Zeo is applied 4 h after the priming treatment with LE in *C. reinhardtii*, *P. sativum* L. and *H. vulgare* L. (design 3). Our data illustrate that pretreatment with LE that induces small level of DNA damage can trigger an AR measured as decrease of SSBs, DSBs, tail moment, MN and MwA. These results contribute to the hypothesis that small but statistically significant level of damages can trigger an AR [11] and also confirm our previous finding that acceleration of DNA rejoing is involved in the formation of an AR [11,13,27].

### 2.5. Modulation of the Clastogenic Effect of Zeocin in *H. vulgare* L.

**Figure 8:**
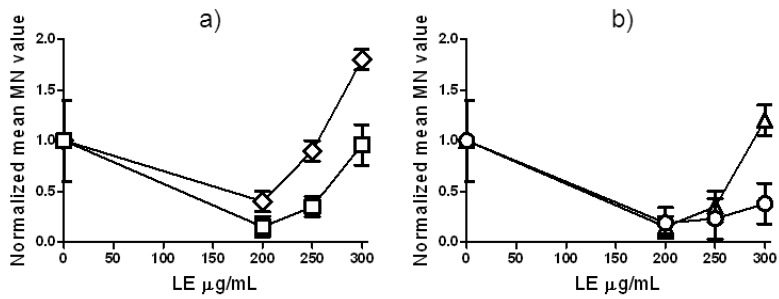
Frequency of micronuclei (MN) in *H. vulgare* L. cells using different experimental designs for treatment with LE (50–300 μg/mL) and Zeo (300 μg/mL). Panel (**a**) diamonds: combined treatment with LE and Zeo (experimental design 1); squares: Zeo-treatment given immediately (without intertreatment time) after LE-treatment (experimental design 2); panel (**b**) triangles: Zeo-treatment given 2 h after priming treatment with LE (experimental design 3) and circles: Zeo-treatment given 4 h after priming treatment with LE (experimental design 3). Where no error bars are evident, errors were equal to or smaller than the symbols.

The same experimental designs and two endpoints were used to evaluate how LE can modulate clastogenic effect of Zeo in *H. vulgare* L. Micronuclei test demonstrates statistically significant increase of MN after the treatment with mixture of LE (300 μg/mL) and Zeo (experimental design 1, Figure 8a). 

LE concentration of 300 μg/mL applied 4 h before treatment with Zeo leads to a decreased level of MN, suggesting some protective effect of LE (experimental design 3, Figure 8b). We show that LE-treatment results either in only a very weak DNA-damaging and clastogenic effects or an absence a significant effect. This suggests that LE behaves similarly to other agents that can act as both clastogens and/or as anticlastogens [24,35]. Such contradictory biological properties of LE could possibly be related to the polyphenolic structure of some LE constituents and partly due to the presence of flavonoids that have been found to possess both prooxidant and antioxidant potential, suggesting their possible dual role in mutagenic/antimutagenic, clastogenic/anticlastogenic and carcinogenic/anticarcinogenic effects [29,36,37]. Niering *et al.* [38] found that high doses of kaempferol can induce DNA damage and apoptosis in rat H4IIE cells and speculated that for some of the intrinsic effects of kaempferol, time and dose requirements are different from those at which its protective effects have been observed. 

**Figure 9:**
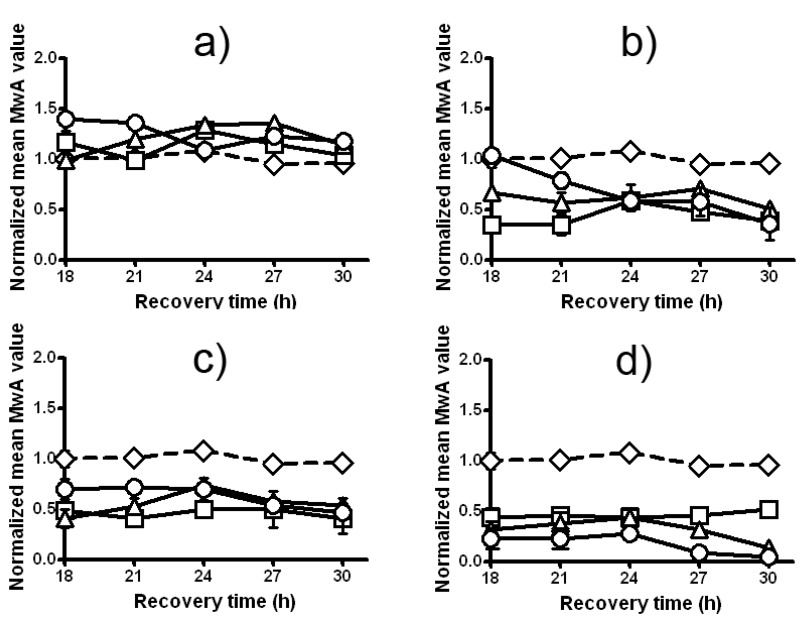
Induced chromatid aberrations in root tips meristem cells of *H. vulgare* L. following different experimental designs; (**a**) combined treatment with LE and Zeo (experimental design 1); (**b**) Zeo-treatment given immediately (without intertreatment time) after LE-treatment (experimental design 2); (**c**) Zeo-treatment given 2 h after priming treatment with LE (experimental design 3); and (**d**) Zeo-treatment given 4 h after priming treatment with LE (experimental design 3). Diamonds represent Zeo 300 µg/mL; squares represent LE 200 µg/mL; triangles represent LE 250 µg/mL and circles represent LE 300 µg/mL. Error bars represent standard deviation of mean values. Where no error bars are evident, errors were equal to or smaller than the symbols.

On the basis of chromatid aberration test no significant differences (P > 0.05) are revealed in experimental design 1 (Figure 9a). A significant decrease of Zeo clastogenic effects is found when Zeo is given immediately after the treatment with LE (experimental design 2, Figure 9b). The largest anticlastogenic effect is reached when 4 h intertreatment time between LE and Zeo is given (experimental design 3, Figures 9c,d). 

**Figure 10:**
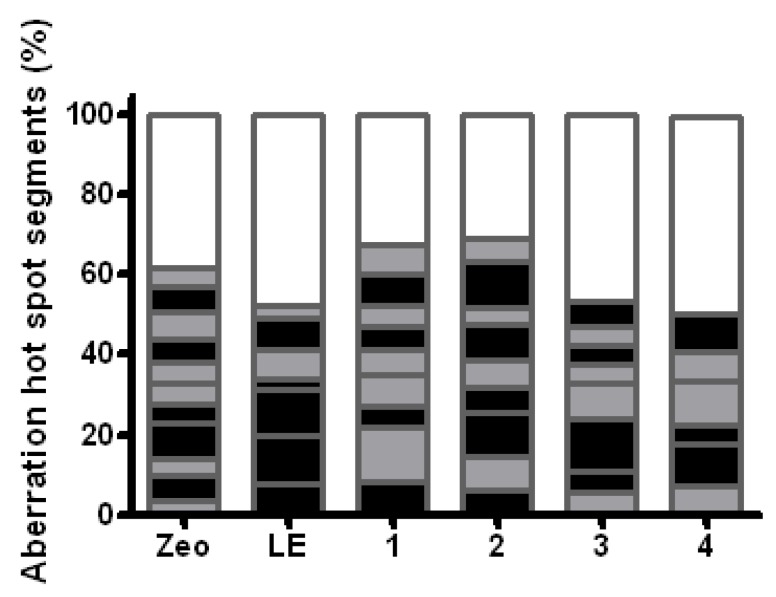
The effect of different experimental designs for treatment with LE and Zeo on the distribution of aberration “hot spots” in root tips meristem cells of *H. vulgare* L. The stripe of the columns demonstrates the number and the localization of “hot spot” segments after specific treatment procedures. Light gray stripes represent proximal “hot spot” breakpoints, black stripes represent terminal “hot spot” breakpoints and white stripes represent “none hot spot” aberration break points. LE priming treatment 300 µg/mL, Zeo test dose 300 µg/mL; 1. combined treatment LE and Zeo (experimental design 1); 2. Zeo-treatment given immediately (without intertreatment time) after LE-treatment (experimental design 2); 3. Zeo-treatment given 2 h after the priming treatment with LE (experimental design 3), 4. Zeo-treatment given 4 h after the priming treatment with LE (experimental design 3).

It is known that aberration *hot spots* represent chromosome segments that are more sensitive to specific clastogens as expected on the basis of the segment lengths. It is found that 11 aberration *hot spots* are induced as a result of a single Zeo-treatment and only seven as a result of single LE-treatment (Figure 10). When analyzing induced *hot spots* it should be mentioned that nine aberration hot spots have been generated when treatment with mixture containing LE (300 μg/mL) and Zeo has been applied (Figure 10, column 1). These *hot spots* are mainly located in the proximal regions of chromosomes. The number of aberration *hot spots* is significantly reduced (P ≤ 0.001) when priming LE-treatment (300 μg/mL) is carried out 4 h before the test treatment with Zeo (Figure 10, column Zeo compared with column 4). In this case six aberration *hot spots* are observed. 

Similar studies concerning the modulation of genotoxicity by phytochemicals were performed by other researchers [7,8]. Nikolić *et al.* [7] revealed that monoterpenes in higher concentrations could induce DNA lesions that were either direct strand breaks or converted into strand breaks during the repair process or the comet-assay procedure. Monoterpenes, like constituents of LE, were inductors of an adaptive response/hormesis phenomenon. 

The adaptive response is now generally accepted as a real and reproducible biological phenomenon, being highly generalized and independent of biological model, end-point measured and chemical/physical stressor applied [39]. Shaugnessy *et al.* [8] found out that dietary antimutagens vanillin and cinnamaldehyde induced DNA breaks that induced recombination repair and DSBs rejoining. Analyzing the kinetics of DNA breaks rejoining induced by Zeo-treatment given 4 h after the priming treatment with LE in *C. reinhardtii* (Figure 6c) and *P. sativum* L. (Figure 7c) we suggest that *Lilium* extract-induced AR to Zeo involves up-regulation of DSBs rejoining in both *C. reinhardtii* and *P. sativum* L. cells. Finally, we can conclude that modulation activity of LE on Zeo mutagenic and clastogenic effects depends not only on concentrations used but also on experimental designs and varies from synergistic to protective with respect to DNA damage.

## 3. Experimental

### 3.1. Extraction Procedure

Bulbs of *Lilium candidum* L. were collected in Bratislava, Slovakia. A voucher specimen has been deposited at the Faculty of Pharmacy, Comenius University, Bratislava, Slovak Republic. The extraction procedure has been described in detail by Mučaji *et al.* [40]. Briefly, fresh bulbs of *L. candidum* L. (1.7 kg) have been extracted with 96% EtOH (3× with 4L,, 7 days , laboratory temperature) and concentrated using a vacuum rotary evaporator at a temperature which did not exceed 40 °C, to give 89 g of residue. This crude ethanol *Lilium* extract (LE) has been used for experiments. The resulting crude ethanol extract contained 3.08% of total flavonoids, 12.05% of total polyphenols and 4.75% of tannins respectively, determined according by the Slovak Pharmacopoeia method [41]. Nitrogenous compounds content (0.35%) was determined according to Fathkiev *et al.* [42]. The extract was dissolved in DMSO and diluted in distilled water. For experiments a stock solution 10 mg/mL of extract has been prepared.

### 3.2. Test Systems and Treatments

*Pisum sativum* L. (*Fabaceae*)–cultivar *Sprinter* seeds were surface sterilized with 96% ethanol for 10 min, thoroughly rinsed 3–4 times with tap water, soaked for 1 h in tap water at room temperature and germinated on moist filter paper in Petri dishes for 3–4 days in the dark at 24–26 °C. Roots with 4–5 mm length have been treated with LE (in a range of 10–250 μg/mL) or with Zeo (50–400 μg/mL) for 2 h at room temperature in the dark.

*Chlamydomonas reinhardii* algal cell-wall-less strain *cw15* has been used. We have previously described the strain characteristics, cultivation conditions and the Zeo-treatment [13,27]. LE concentrations in the range from 50 μg/mL to 300 μg/mL and treatment time 2 h have been applied. To prevent repair of induced DSBs all treatments have been performed at t = 4 °C. 

**Figure 11:**
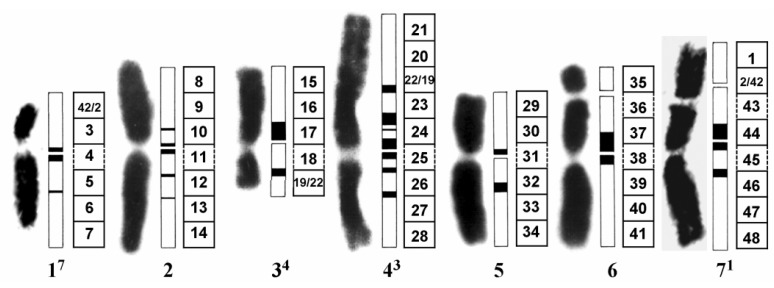
Reconstructed barley karyotype MK14/2034. For analyzing the regional specificity of aberration induction the metaphase chromosomes of *H. vulgare* L. were subdivided into 48 segments. The segments are numbered with respect to their position in the standard karyotype (modified schema based on Künzel and Nicoloff [43]). Black bars in the schematic chromosomes represent heterochromatic regions.

*Hordeum vulgare* L. (*Poaceae*)–reconstructed karyotype (MК 14/2034) (Figure 11) has been obtained as a result of the combination of two simple reciprocal translocations between chromosomes 1 and 7 and chromosomes 3 and 4 [43]. The advantages of this karyotype have been described previously [24,44,45].

For analyzing the regional specificity of aberrations, the metaphase chromosomes of *H. vulgare* L. have been subdivided into 48 segments and each segment numbered with respect to its position in the standard karyotype. The percentage of aberrations in the individual chromosome segments have been calculated based on the total number of aberrations in a several treatment variant over all chromosome segments.

Seeds soaked for 1 h in tap water at room temperature have been germinated on moist filter paper in Petri dishes for 18 h in the dark at 24 °C. The germinated seeds have been treated with 50–300 μg/mL of LE and with Zeo (100–300 μg/mL) for 1 h in the dark. Untreated root tip meristems have been used as a negative control.

### 3.3. Mitotic Index (MI)

MI as a cytotoxicity marker has been evaluated according to the procedure described by Jovtchev *et al*. [46]. MI has been calculated according to the formula MI = A/1000 (where A is a number of dividing cells).

### 3.4. Comet Assay (CA)

A comet assay [47] has been used to measure LE- and Zeo-induced DNA damage (genotoxicity) in *P. sativum* L. In short, a suspension of nuclei prepared from seedling roots has been mixed with agarose and then this mixture has been spread on cold-resistant microscope slides (Superfrost). Lysis and electrophoresis has been performed at alkaline pH. Nuclei have been analyzed at 100× magnification using an Olympus BX51 fluorescence microscope equipped with a U-MNU2 filter and captured by Color View Soft Imaging System Olympus U-CMAD3 and the program AnalySIS 3.0. Images have been analyzed with the image analysis software CometScore™ (TriTec Corporation). The amount of DNA damage has been assessed on the basis of a tail moment (TM) calculated as the product of tail length (from trailing edge) and the fraction of DNA in the comet tail.

### 3.5. Constant Field Gel Electrophoresis (CFGE)

CFGE, used to evaluate the fraction of induced double strand breaks (DSBs) or the fraction of DSBs remaining, has followed the procedure described in detail previously [13,14,27]. The movement of DNA out of the starting wells into the electrophoresis gel has been measured using UV-gel scan and computer analysis of DNA-ethidium bromide fluorescence (Syngene software; GeneTools).

### 3.6. Micronuclei (MN) and Metaphases with Chromatid Aberrations (MwA)

Induced MN and MwA have been used as markers for clastogenicity. MwA and MN have been analyzed and scored according to the procedures described in detail by Jovtchev *et al*. [46].

### 3.7. Experimental Designs

To distinguish between a possible protective action of *Lilium* extract and a damaging one, three types of treatments identified as experimental designs (1-3) have been performed. Experimental design (1) was a treatment with a mixture of LE and Zeo; experimental design (2), a split treatment without any intertreatment time between LE- and Zeo-treatment; and experimental design (3), a split treatment with 2 and/or 4 h intertreatment time between LE- and Zeo-treatment. Priming treatment with LE given prior to a test dose of Zeo is named split treatment. All treatments have been carried out in the dark. Two recovery times have been allowed after the treatment with high Zeo concentrations (test treatment with Zeo). During the intertreatment time, samples have been kept at room temperature in the dark for 2 h and 4 h.

### 3.8. Statistics

Data presented are mean values from at least three experiments performad in *C. reinhardtii* and in *P. sativum* L. Error bars represent standard errors of mean values. Where no error bars are evident, errors have been equal to or less than the symbols. The statistical analysis has been done by GraphPad Prism, t-test and Fisher exact test. One-way ANOVA with Tukey multiple comparison test has been used to compare the mean differences between samples (GraphPad Prism software).

The mean fraction of damage remaining (FDR), tail moment value (TM), micronuclei (MN) and metaphases with chromatid aberration (MwA) after split treatment experiments have been normalized according to Snedecor and Cochran [48], Bryant [49], Chankova and Bryant [27], and Chankova *et al*. [13,50]. The capacity of cells to rejoin DSBs at 2 and 4 h after treatment has been defined as the ratio of the fraction of damage at 0 h to the FDR at 2 and 4 h recovery time, respectively.

The first post-treatment mitosis has been analyzed to determine the percentage of MwA in *H. vulgare* L. Two-tailed Fisher’s exact test has been used for the statistical analysis on the basis of at least 750 cells, *i.e.*, 150 cells per recovery time for MwA’s, 1,000 cells for MN and 300 cells for MI per experiment. An adapted formula [46,51] has been used for a comparison of the upper limit of confidence interval of the expected chromatid aberrations in individual loci.

## 4. Conclusions

To our knowledge, this study provides the first experimental evidence that the clastogenic and genotoxic effects of Zeo in plants could be modulated by *Lilium* extract. The mode of action strongly depends on the experimental design and varies from synergistic to DNA protective, with respect to DNA damage. Pretreatment with LE can not only accelerate DSBs repair in *C. reinhardtii* and *P. sativum* L. but LE in the same experimental design can also decrease the clastogenic effects of Zeo and the level of aberration *hot spots* in *H. vulgare* L. when priming treatment was applied 4 h prior to test concentration. Analyzing the kinetics of SSBs and DSBs rejoining induced by Zeo it was revealed that *Lilium* extract-induced AR to Zeo involves up-regulation of DSBs rejoining in *C. reinhardtii* and *P. sativum* L. cells.
